# Screening for Hypertension Among Older Adults: A Primary Care “High Risk” Approach

**DOI:** 10.4103/0970-0218.62561

**Published:** 2010-01

**Authors:** Jacob John, Jayaprakash Muliyil, Vinohar Balraj

**Affiliations:** Department of Community Health, Christian Medical College, Vellore - 632 002, India

**Keywords:** Screening, hypertension, primary care

## Abstract

**Background:**

Recommendations for early detection and management of elevated blood pressure through opportunistic clinic-based screening may be inadequate for the rural population in India as access to health facilities is limited.

**Materials and Methods:**

Sixteen Health Aides (trained primary care workers) were trained to measure blood pressure using a standardized training procedure. Six of those assessed competent in initial evaluation were allotted a stratified random sample of about 150 persons each, 50 years or over, in the village under their care to measure blood pressures during their regular scheduled visits.

**Results:**

14/16 of the health aides (83%) met the stipulated criteria for the simulation study using a module from British Hypertension Society. In the field survey of 920 individuals where 20% of the population was evaluated by a blinded investigator, the weighted Kappa for agreement, using normal, pre-hypertension and hypertension as categories, ranged between 62% and 89%. Only 75/286 (25%) of those detected to be hypertensive knew their status prior to the study. All those detected with hypertension were referred to a physician at a referral facility. 70% of those referred were evaluated at the referral facility and 64% of them initiated on treatment for hypertension within 3 months.

**Conclusion:**

Using primary care workers to screen for hypertension through the model suggested here will ensure that the population over 50 years of age will be screened once every 2 years without burdening the worker. This screening process will enable the health system to identify and cater to needs of this vulnerable population.

## Introduction

The World Health Report 1999(1) estimates that in 1998, 85% of the cardiovascular burden arose from the low-and middle-income countries. It is estimated that by the year 2025, the majority of the elderly people worldwide will reside in developing countries.(2) Developing countries are thus likely to face an enormous burden of vulnerable elderly population who are predisposed to chronic non-communicable diseases.

Hypertension is one of the most important treatable causes of mortality and morbidity in the elderly population. Epidemiological studies on hypertension suggest that less than half the hypertensive persons in a population know they have elevated blood pressure. The CUPS(3) study suggests that the “rule of halves” is still valid in Indian population making control of hypertension in the population inadequate. The health seeking characteristics in rural India, driven largely by economic and socio-cultural considerations, preclude large proportion of the older persons from formal health care access unless a serious ailment intervenes. Over half the outpatient consultations are with indigenous and private practitioners(4) where regular screening for hypertension is not practiced. Clinic-based (opportunistic) screening of hypertension is unavailable to those in these settings. A community-based screening can improve the detection of this ‘silent killer’ and thereby provide a point of intervention. ‘Absolute cardiovascular risk status’-based approach for treating hypertension implies that treatment should be prioritized to those individuals who have multiple risk factors for CVD. The older population form an ideal intervention group because other risk factors are more common among them as well.

In countries with limited healthcare resources such as India, the use of trained primary care health workers for screening for hypertension and monitoring treatment would, we believe, improve the detection and compliance to treatment. A study was conducted to assess whether Health Aides, similar in training and educational qualifications to the Village Health Nurse, can be sufficiently trained to measure blood pressure without substantially increasing workload.

## Materials and Methods

Community Health Department of a medical college in Tamil Nadu provides primary health care for a population of 108,000. Sixteen Health Aides, who provide services to a 5000 population each, were trained to conduct hypertension screening. Training was done in five sessions, each lasting 2 h, at the secondary care hospital when the health aides came for reporting activities. For training in Koratkoff sounds and correlation with the falling mercury column, a specially designed module from the British Hypertension Society (BHS)(5) was utilized. This was projected on screen with LCD projector, while the Health Aides listened to the sound on headphones. Twelve such modules tested the Health Aides for various conditions including auscultatory gap, extraneous noise, and atrial fibrillation. Six Health Aides among those who graduated the training were randomly selected and provided with a mercury sphygmomanometer (Diamond^®^ Deluxe) and a stethoscope (Microtone^®^) with both a bell and diaphragm. An age-stratified random list of about 150 persons each between 50 and 80 years selected from the Health Aide's geographic area of work was also provided; Health Aides were required to screen the persons in the list during their routine visits to the villages for primary care. They were required to complete their screening target within 12 weeks. They were permitted to choose the order and rate at which they would screen the study population. An investigator blinded to their findings followed his schedule and checked the blood pressure on 20% of subjects using the same protocol non-concurrently. Those who on screening were found to be hypertensive were referred to the secondary care hospital, or if that were not possible, to the primary care physician during a monthly village visit. At the secondary care hospital, protocols were drawn up for evaluation of hypertension and starting on treatment where considered necessary. For the purpose of estimating prevalence of hypertension, all reported hypertensive persons irrespective of their current blood pressure status were included as hypertensive along with newly detected hypertensive persons. Data collected were entered on Microsoft Excel and analyzed with SPSS (Version 12.0). Statistical methods used in the analysis were Chi-square, weighted Kappa, and Pearson's correlation coefficient.

## Results

The age of the Health Aides ranged between 29 and 57 years. The mean age was 46 years. The Health Aides on an average completed 11 years of schooling. The average score at the school leaving examination for was 55% marks. In the audiovisual simulation using the BHS modules 14/16 (83%) of the Health Aides acquired adequate competence in identifying Korotkoff sounds and correlating it to the readings on a video clip of falling mercury column on the screen. In the second level of training where the health-aides measured blood pressures concurrently with an investigator on 11 volunteers, the mean difference in blood pressure measurements was 0.835, with a pooled SD of 7.186 (95% tolerance interval - 13.54 to 15.17). Pearson's correlation coefficient r = 0.973 (significant at 0.01 level) for correlation of systolic blood pressure measurement between investigator and Health Aides as a group suggests a good correlation.

In the community screening where six health aides participated, the weighted kappa between health aide and the investigator ranged from 62% to 89%. Normal blood pressure, pre-hypertension, and hypertension were used as the categories for calculating weighted kappa. The inter-observer agreement between each Health Aide and an investigator is summarized in [Table 1].

**Table 1:** Inter-observer (health aide vs investigator) agreement of blood pressure readings of study population measured in field conditions

HA number	Systolic		Diastolic	
		
	Kappa	Standard error	Kappa	Standard error
17	0.852	0.171	0.804	0.174
15	0.614	0.155	0.617	0.171
13	0.896	0.163	0.876	0.176
7	0.816	0.186	0.731	0.186
14	0.828	0.187	0.664	0.185
5	0.854	0.213	0.943	0.216

Of the 920 persons over the age of 50 years screened for hypertension, 33% (309 persons) had pre-hypertension (systolic BP 120-139 mmHg or diastolic BP 80-89 mmHg) as defined in *The Seventh Report of the Joint National Committee on* Prevention, Detection, Evaluation, and Treatment of High Blood Pressure (JNC7).(6) The prevalence of hypertension was 28%. The prevalence of hypertension increased from 25% in those below 60 to 37% in those above 70 years [Chi-square for trends χ^2^ = 14.829 (*P* = 0.001)]

Only 25% (75/286) of those found to have hypertension were previously aware that they were hypertensive. Only 21% (16/75) of those who knew they were hypertensive had blood pressures under control. 70% of those newly detected to have elevated blood pressure were evaluated by a physician at the referral facility and 64% of them were initiated on treatment for hypertension within 3 months. 30% of those with elevated blood pressures did not follow referral advice or refused to start treatment. Of those seen by a physician, 32% did not require medical management of blood pressure and were advised lifestyle changes where required.

## Discussion

Older people tend to have higher absolute cardiovascular risk status, as there is an unambiguous trend towards higher blood pressure with increasing age and are therefore likely to gain substantially by reduction of blood pressure.(7,8)

The emerging social neglect and inequity in this group makes the older age groups a vulnerable population who are not covered by opportunistic clinic-based screening. In the context of primary health care, they constitute a high priority group. Treatment is now available at affordable prices and therefore identification of hypertension among them and initiation of therapy will form a vital component of integrated management of cardiovascular diseases.

The WHO's CVD risk management package provides for screening for hypertension using primary care workers.(9) Primary Care Workers have in this study been successfully trained to screen for hypertension. Screening for hypertension based on WHO CVD risk management package have been similarly found successful in detecting and decreasing elevated blood pressure.(10) The rate of coverage used in the study ensures that over a 2-year period, all those between 50 and 75 years in that geographic area would receive one round of screening, without upsetting routine work. The study demonstrates that by prioritizing screening to include those with highest risk of cardiovascular events and poorest access to health care, primary care workers can provide a crucial health facility without increasing their workload substantially. Screening this age subset alone might cause the program to miss a substantial number of younger hypertensive individuals and the age group for screening must consider population structure and workload of health worker. We used weighted kappa as a measure of inter observer agreement. Since this is a screening at the community the ability to detect normal, pre-hypertensive, and hypertensive individuals is the priority. We did not concurrently measure blood pressure of subjects in the community, as this was neither logistically feasible nor desirable, as it would interfere with the normal functioning of health aides.

This study also demonstrated that hypertension was widely prevalent among older persons. 75% of those with hypertension were unaware of its presence. A community-based hypertension screening program targeting older adults, such as the one described here could help detect persons with this ‘silent killer’ early and initiate therapy. While this study demonstrates a community-based screening program for control of hypertension, it is essential to study further how those detected to be hypertensive can be motivated to seek care and comply with therapy. Furthermore, we recognize that health workers may not always be as motivated and have access to details of the older population to conduct a selective screening process. These concerns however are not insurmountable, as we gear to respond to emerging public health issues.
